# NHERF1, a novel GPER associated protein, increases stability and activation of GPER in ER-positive breast cancer

**DOI:** 10.18632/oncotarget.10713

**Published:** 2016-07-19

**Authors:** Ran Meng, Qiong Qin, Ying Xiong, Yan Wang, Junfang Zheng, Yuan Zhao, Tao Tao, Qiqi Wang, Hua Liu, Songlin Wang, Wen G. Jiang, Junqi He

**Affiliations:** ^1^ Department of Biochemistry and Molecular Biology, Capital Medical University, Beijing, China; ^2^ Beijing Key Laboratory for Tumor Invasion and Metastasis, Beijing International Cooperation Base for Science and Technology on China-UK Cancer Research, Beijing, China; ^3^ Molecular Laboratory for Gene Therapy and Tooth Regeneration, Capital Medical University School of Stomatology, Beijing, China; ^4^ Cardiff China Medical Research Collaborative, Cardiff University School of Medicine, Cardiff, United Kingdom

**Keywords:** G protein-coupled receptor, EBP50, protein-protein interaction, protein degradation, carcinogenesis

## Abstract

G protein-coupled estrogen receptor (GPER) plays an important role in mediating the effects of estradiol. High levels of GPER have been implicated to associate with the malignant progress of invasive breast cancer (IBC). However, the mechanisms by which GPER protein levels were regulated remain unclear. In this study, PDZ protein Na^+^/H^+^ exchanger regulatory factor (NHERF1) was found to interact with GPER in breast cancer cells. This interaction was mediated by the PDZ2 domain of NHERF1 and the carboxyl terminal PDZ binding motif of GPER. NHERF1 was demonstrated to facilitate GPER expression at post-transcriptional level and improve GPER protein stability by inhibiting the receptor degradation via ubiquitin-proteasome pathway in a GPER/NHERF1 interaction-dependent manner. In addition, GPER protein levels are positively associated with NHERF1 protein levels in a panel of estrogen receptor (ER)-positive breast cancer cells. Furthermore, analysis of clinical IBC data from The Cancer Genome Atlas (TCGA) showed no significant difference in GPER mRNA levels between ER-positive IBC and normal breast tissues. However, gene set enrichment analysis (GSEA) showed that GPER signaling is ultra-activated in ER-positive IBC when compared with normal and its activation is positively associated with NHERF1 mRNA levels. Taken together, our findings identify NHERF1 as a new binding partner for GPER and its overexpression promotes protein stability and activation of GPER in ER-positive IBC. Our data indicate that regulation of GPER stability by NHERF1 may contribute to GPER-mediated carcinogenesis in ER-positive IBC.

## INTRODUCTION

G protein-coupled estrogen receptor (GPER), also called GPR30, is a newly found estrogen receptor belonging to G protein-coupled receptor (GPCR) family [1, 2]. GPER is extensively expressed in numerous tissues such as breast, uterus, ovary and brain. It has been reported to play physiological roles in regulating the functions of cerebral, endocrine and reproductive system *etc.* [3, 4]. Estrogen binding with GPER can activate multiple down-stream effectors, including the production of cAMP [5], calcium mobilization [6, 7], PI3K/Akt and Hippo/YAP/TAZ pathway activation [7–9] and EGFR/MAPK transactivation [7, 10]. GPER has also been reported to contribute to pathological responses such as cancer cells proliferation, migration and invasion, especially breast cancer development [3, 10].

Recently, high protein levels of GPER have been reported to positively correlate with increased tumor size, distant metastasis and poor prognosis of breast cancer [11, 12]. *In vivo* study from transgenic mice tumor model showed that deletion of GPER reduced the size of mammary tumors and lung metastasis, indicating that GPER is critical in breast tumor growth and distant metastasis [13]. The detailed molecular mechanisms that lead to up-regulation of GPER protein remain unclear. Total protein levels could be regulated both from transcriptional and post-translational levels. Hypoxia, epidermal growth factor and Insulin-like growth factor-I have been implicated to increase GPER expression in transcriptional level [14–16]. Meanwhile, GPER protein has been reported to display low stability with a high turnover rate [17]. However, the molecular mechanisms underlying the regulation of GPER stability remain to be further elucidated.

The stability of some GPCRs could be regulated by binding with PDZ domain containing proteins. Our previous study showed that interaction with postsynaptic density-95 protein (PSD-95) enhanced Mas protein level by increasing its stability [18]. Another PDZ protein, CAL, inhibited degradation of metabotropic glutamate receptor 5a and β1-adrenergic receptor via interaction with the receptors [19, 20]. GPER is also a member of GPCRs and possesses a PDZ-binding motif at its C-terminal. PDZ proteins PSD95 and synaptic associated protein 97 (SAP97) could bind with GPER and regulate the signaling and trafficking of GPER in hippocampal dendritic spines or HEK-293 cells respectively [21–23]. However, neither GPER/PDZ protein interaction in breast cancer cells nor the modulation of GPER stability by PDZ proteins has been reported so far. Na^+^/H^+^ exchanger regulatory factor (NHERF1) is a PDZ protein with well reported roles in the regulation of stability of its binding partners [24, 25]. NHERF1 is highly expressed in breast carcinoma and positively correlated with tumor size and grade, especially in estrogen receptor (ER)-positive breast carcinoma [26–28]. Our previous study demonstrated that NHERF1 can interact with PTEN and enhance PTEN protein stability [24]. Interestingly, GPER possesses a PDZ binding motif similar to PTEN (S/T-X-V). Thereafter, we speculate that NHERF1 may also interact with GPER and regulate its expression level.

In this study, we found that NHERF1 interacted with GPER via the PDZ2 domain of NHERF1 and the C-terminal of GPER. Further, NHERF1 enhanced the stability of GPER through inhibition of GPER ubiquitination. By analyzing clinical breast cancer data from TCGA, we also found a positive correlation between NHERF1 expression and GPER pathway activation in ER-positive breast cancer. These findings provide a new insight into the regulatory mechanism of the GPER protein by NHERF1 in breast cancer cells.

## RESULTS

### NHERF1 is identified as a novel GPER-associated protein

**Figure 1:**
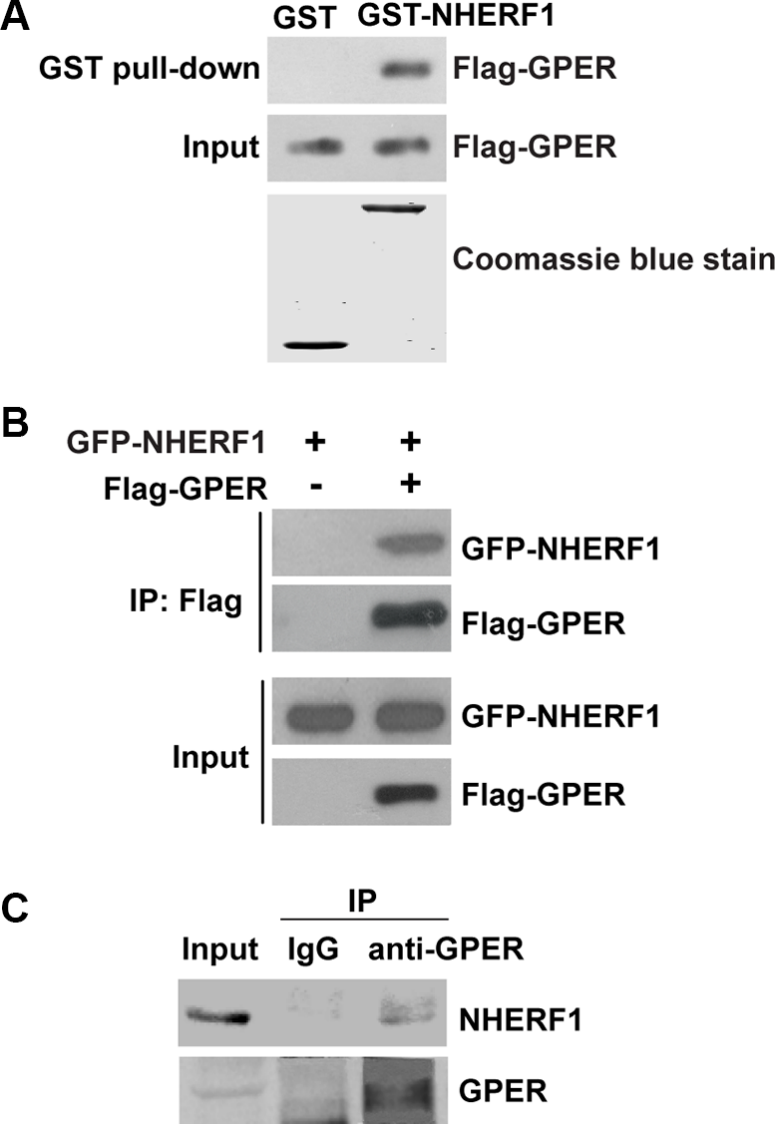
NHERF1 is identified as a novel GPER-associated protein (**A**) GPER interacts with NHERF1 *in vitro*. Lysates of HEK-293 cells that stably transfected with Flag-GPER (HEK-GPER) were incubated with GST or GST-NHERF1, followed by Western blotting using anti-Flag antibody. GST and GST-NHERF1 fusion proteins were stained by Coomassie blue dye (lower panel). (**B**) Cellular association of full-length Flag-GPER with GFP-NHERF1 in HEK-293 cells. HEK-293 cells were transfected with GFP-NHERF1 in the presence or absence of Flag-GPER. The cells were then lysed and immunoprecipitated using anti-Flag affinity gel. The immunoprecipitate samples (IP) and whole cell lysates (Input) were analyzed by Western blotting with anti-Flag or anti-GFP antibodies. (**C**) GPER associates with NHERF1 in MCF-7 cells. The lysates of MCF-7 cells were subjected to immunoprecipitation using anti-GPER antibody. Lysates and immunoprecipitated samples were probed with anti-GPER or anti-NHERF1 antibodies to detect the presence of GPER and NHERF1. Blots are representative of three to five independent experiments. Input, whole cell lysates; IP, immunoprecipitation.

To test the possibility of GPER and NHERF1 interaction, we first performed GST pull-down analysis. GST-NHERF1 fusion protein was used to pull down the lysates of HEK-293 cells stably transfected with Flag-GPER (HEK-GPER). As shown in Figure 1A, Flag-GPER was robustly detected in GST-NHERF1 pull-down fraction, whereas no detectable immunoreactivity was observed in the GST control pull-down complex (Figure 1A), indicating that GPER interacts with NHERF1.

To verify this interaction in cells, HEK-293 cells were transfected with GFP-NHERF1 in the presence or absence of Flag-GPER. Immunoprecipitation (IP) of Flag-GPER was followed by Western blotting and a strong NHERF1 signal was found in Flag-GPER-IP complex (Figure 1B). These results were further verified by co-IP of endogenous NHERF1 with endogenous GPER in MCF-7 breast cancer cells (Figure 1C). Taken together, these data indicate that NHERF1 is a novel binding protein of GPER.

### C-terminal of GPER binds to the PDZ2 domain of NHERF1

**Figure 2:**
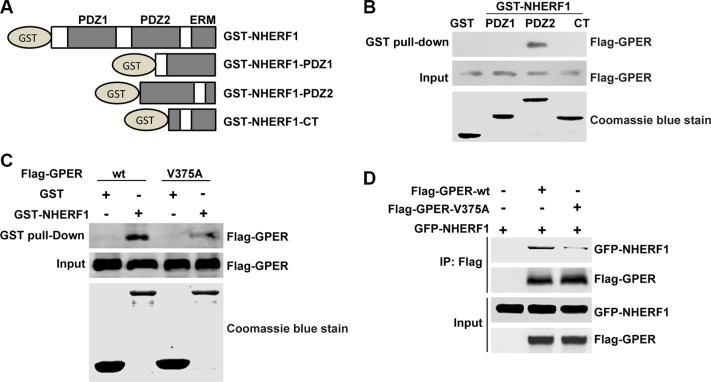
Interaction of NHERF1 and GPER is mediated via the C-terminal of GPER and the PDZ2 domain of NHERF1 (**A**) Schematic diagram of GST-NHERF1 fusion proteins used in GST-pull down experiments. (**B**) GPER interacts specifically with the PDZ2 domain of NHERF1. Lysates of HEK-GPER were incubated with GST or GST fusion proteins containing different domains of NHERF1. Precipitates were subjected to Western blotting with anti-Flag antibody. Coomassie blue staining showed equal loading of the fusion proteins (bottom panels). (**C**) Association of NHERF1 and GPER via C-terminal of GPER. Equal amounts of purified GST or GST-NHERF1 fusion protein beads (lower panel) were used to pull down lysates from HEK-293 cells that transfected with Flag-GPER-wt or Flag-GPER-V375A respectively. Precipitates were detected by Western blotting using anti-Flag antibody. (**D**) The point mutation of GPER (GPER-V375A) abolishes the interaction between GPER and NHERF1 in cells. HEK-293 cells were either transfected with Flag-GPER-wt or Flag-GPER-V375A together with or without GFP-NHERF1. Cell lysates were then immunoprecipitated using anti-Flag affinity gel. Immunoprecipitated samples were subjected to Western blotting using anti-Flag or anti-GFP antibodies. The expression levels of Flag-GPER-wt and Flag-GPER-V375A were adjusted to similar levels through transfection with different amounts of the respective constructs. The experiments were repeated at least three times.

NHERF1 possesses two non-identical tandem PDZ domains and a carboxyl-terminal (CT) ezrin-binding domain [29]. To identify the specific domain of NHERF1 responsible for this interaction, each domain of NHERF1 was subjected to GST pull-down assay separately (Figure 2A). Immunoblotting results showed that GPER robustly bound to PDZ2, but not PDZ1 or CT domain of NHERF1 (Figure 2B). To further determine interaction sites of GPER, the valine^375^ residue of GPER, which is the main determinant for its binding to PSD-95 [21], was mutated to alanine (GPER-V375A). A further GST pull-down experiment was performed using GST-NHERF1. As shown in Figure 2C, the point mutation of GPER dramatically abolished its binding capacity to NHERF1, revealing the requirement of the C-terminal of GPER for the GPER/NHERF1 interaction. Consistently, results of reverse co-IP showed that cellular interaction with NHERF1 also needed intact C-terminal of GPER (Figure 2D). Taken together, these results suggest that the interaction between GPER and NHERF1 is specifically mediated by the NHERF1-PDZ2 domain and the C-terminal of GPER.

### GPER co-localizes with NHERF1 in breast cancer cells

**Figure 3:**
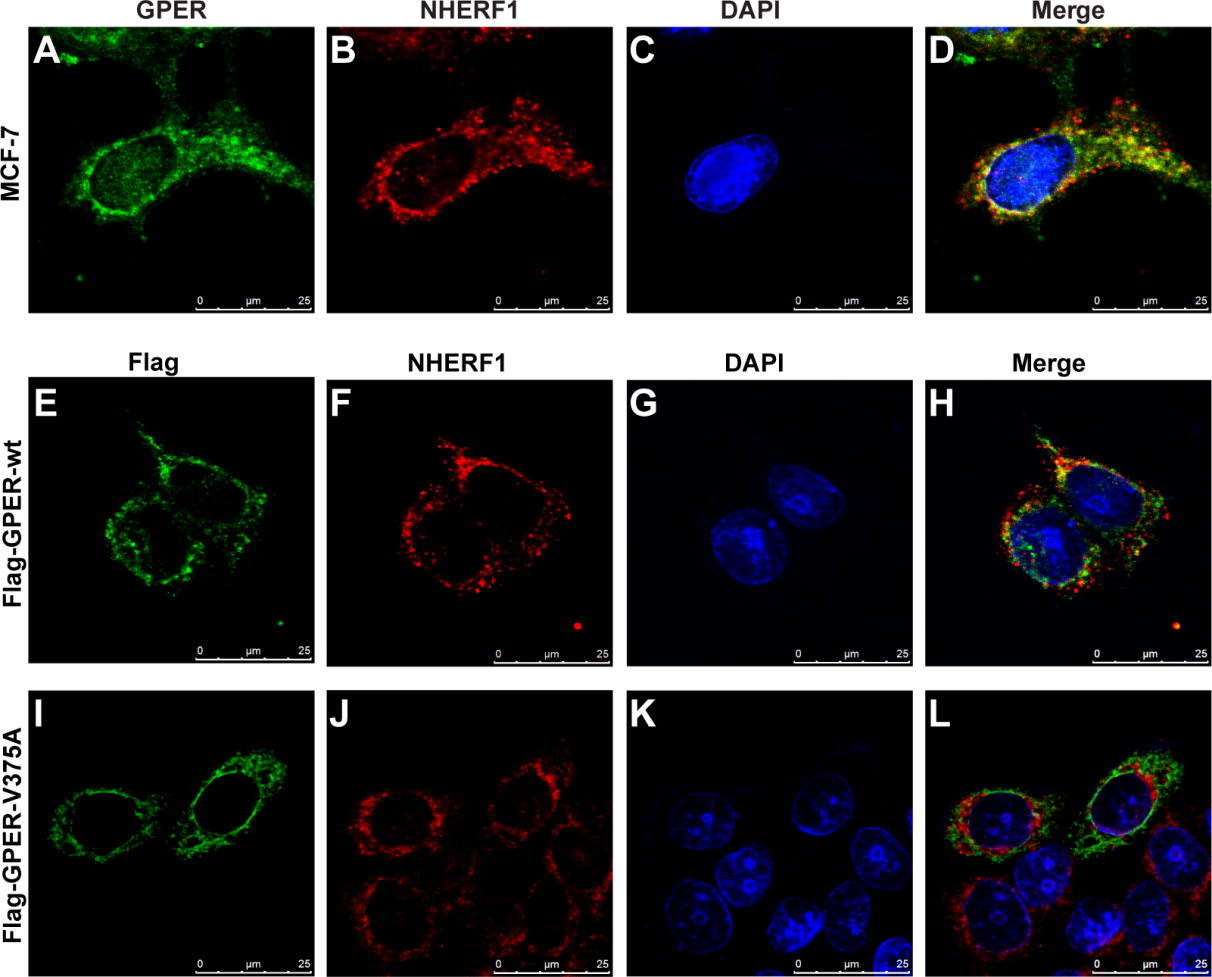
NHERF1 co-localizes with GPER in MCF-7 cells (**A**–**D**) Endogenous GPER and NHERF1 are co-localized in MCF-7 cells. MCF-7 cells were stained with anti-GPER (A) and anti-NHERF1 (B) antibodies followed by visualized using Alexa 488- and Alexa 594-conjugated secondary antibodies. Co-localization of GPER and NHERF1 is shown in yellow color in merged image (D). (**E**–**H**) Wild type GPER co-localizes with NHERF1 in MCF-7 cells. MCF-7 cells were transfected with Flag-GPER-wt, and then incubated with anti-Flag (E) and anti-NHERF1 (F) primary antibodies followed by staining with Alexa 488- and Alexa 594-conjugated secondary antibodies. Nucleuses were stained with DAPI (G). Co-localization of Flag-GPER-wt and NHERF1 is shown in yellow color in panel (H). (**I**–**L**) Mutation in the C-terminal of GPER abolishes its co-localization with NHERF1 in MCF-7 cells. MCF-7 cells were transfected with Flag-GPER-V375A and then stained using anti-Flag (I) and anti-NHERF1 (J) antibodies. Nuclei were detected with DAPI staining (K). Less co-localization of Flag-GPER-V375A and NHERF1 is detected following merging of the two individual images (L).

To further confirm GPER/NHERF1 interaction in intact cells, MCF-7 cells were double stained using anti-GPER and anti-NHERF1 antibodies. Our data showed strong co-localization of GPER with NHERF1 intracellularly by forming multiple spots in cytoplasm, especially areas surrounding the nucleus in MCF-7 cells (Figure 3A–3D). To examine the specificity of this co-localization, Flag-GPER-wt or Flag-GPER-V375A were transfected into MCF-7 cells respectively. Cells were then stained with anti-Flag and anti-NHERF1 antibodies. As shown in Figure 3H, a significant fraction of Flag-GPER-wt co-localized with NHERF1. In contrast, the co-localization between Flag-GPER and NHERF1 was dramatically diminished when their interaction was abrogated by mutation of the C-terminal valine to alanine (V375A) (Figure 3L). Similar results were also detected in T47D breast cancer cells (data not shown). These data, combined with the findings from the co-IP (Figures 1B–1C, 2D) and GST pull-down (Figure 2C) experiments, indicate that GPER interacts with NHERF1 in breast cancer cells and this interaction requires intact C-terminal of GPER.

### NHERF1 increases the expression level of GPER protein at the post-translational level

**Figure 4:**
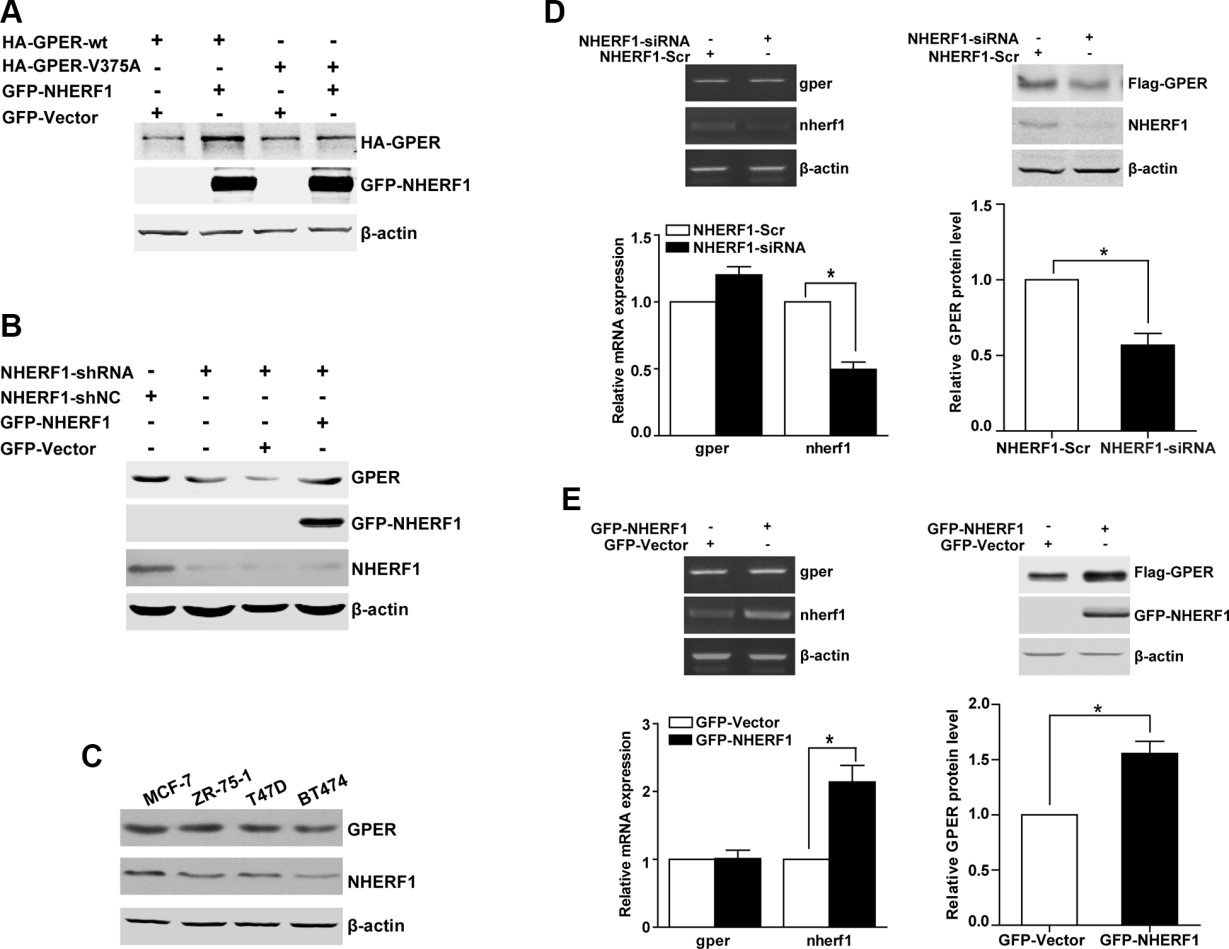
NHERF1 enhances the level of the GPER protein at the post-transcriptional level (**A**) The protein level of GPER-wt is increased when NHERF1 is overexpressed. HEK-293 cells were transiently transfected with equal amount constructs of HA-GPER-wt or HA-GPER-V375A in the presence or absence of GFP-NHERF1. Protein levels of GPER and NHERF1 were detected by Western blotting using anti-HA and anti-GFP antibodies respectively. (**B**) NHERF1 enhances GPER protein level in breast cancer cells. MCF-7 cells were stably transfected with shNC or shNHERF1 (lane 1 and lane 2), and the expression of NHERF1 was rescued by transiently transfected the cells with GFP-Vector or GFP-NHERF1 (lane 3 and lane 4). The cell lysates were then tested by Western blotting using anti-GPER, anti-GFP or anti-NHERF1 antibodies. (**C**) Correlation of endogenous NHERF1 and GPER protein levels in ER-positive breast cancer cell lines. Lysates from four ER-positive breast cancer cell lines were analyzed by Western blotting by using anti-GPER or anti-NHERF1 antibodies. (**D**) Knock-down of NHERF1 expression decreases GPER expression at the post-transcriptional level. HEK-GPER cells were transiently transfected with siNHERF1 or scrambled sequence (Scr) respectively. Total RNA of the cells was isolated using Trizol reagent and the mRNA levels of GPER, NHERF1, and β-actin were then analyzed by RT-PCR (Left panel). Total cell lysates were subjected to Western blotting and detected with anti-Flag, anti-NHERF1, and anti-β-actin antibodies (Right panel). (**E**) Overexpression of NHERF1 dramatically increases GPER expression at post-transcriptional level. HEK-GPER cells were transiently transfected with GFP-NHERF1. The mRNA and protein levels of GPER, NHERF1, and β-actin were analyzed by RT-PCR (Left panel) and Western blotting (Right panel) as described in panel D, respectively. The experiments were repeated at least three times, with values within each experiment normalized with β-actin and analyzed by GraphPad Prism 5. Histogram represents average value of relative GPER protein levels (*p < 0.05). Data were presented as means ± SEM.

We next explored the potential functional significance of the GPER/NHERF1 interaction. In the immunoprecipitation studies, we noted that the expression level of GPER-wt was significantly higher than that of the GPER-V375A mutant when the same amount of plasmid was co-transfected with GFP-NHERF1. The expression of Flag-GPER-wt and Flag-GPER-V375A were adjusted to similar levels through transfection with different amounts of the respective constructs, which would allow comparison of NHERF1 association with the wild-type receptor or the mutant one (Figure 2D). In further experiments, we found that co-expression of NHERF1 could positively regulate total protein expression level of GPER-wt, but not GPER-V375A (Figure 4A). Thus, it appears that GPER/NHERF1 interaction complex may be involved in the regulation of GPER expression level. To further confirm these results in breast cancer cells, NHERF1 was knocked down in MCF-7 cells using shNHERF1. As expected, inhibition of NHERF1 resulted in a significant down-regulation of GPER protein (Figure 4B, lanes 1, 2). Rescue experiment in which NHERF1 is re-introduced in MCF-7-shNHERF1 cells showed that GPER protein levels were recovered (Figure 4B, lanes 3, 4). Consistent with what we found in MCF-7 cells, similar effects of NHERF1 on GPER expression were also found in T47D and SK-BR-3 cells after NHERF1 overexpression or knock-down (Figure S1). In addition, we further examined the correlation between endogenous proteins levels of NHERF1 and GPER in a panel of ER-positive breast cancer cell lines (MCF-7, T47D, ZR-75-1 and BT474 cells). We found that cells expressing higher protein levels of NHERF1 exhibited a higher protein level of GPER, suggesting a positive correlation between expression of GPER and NHERF1 in ER-positive breast cancer cells (Figure 4C).

**Figure 5:**
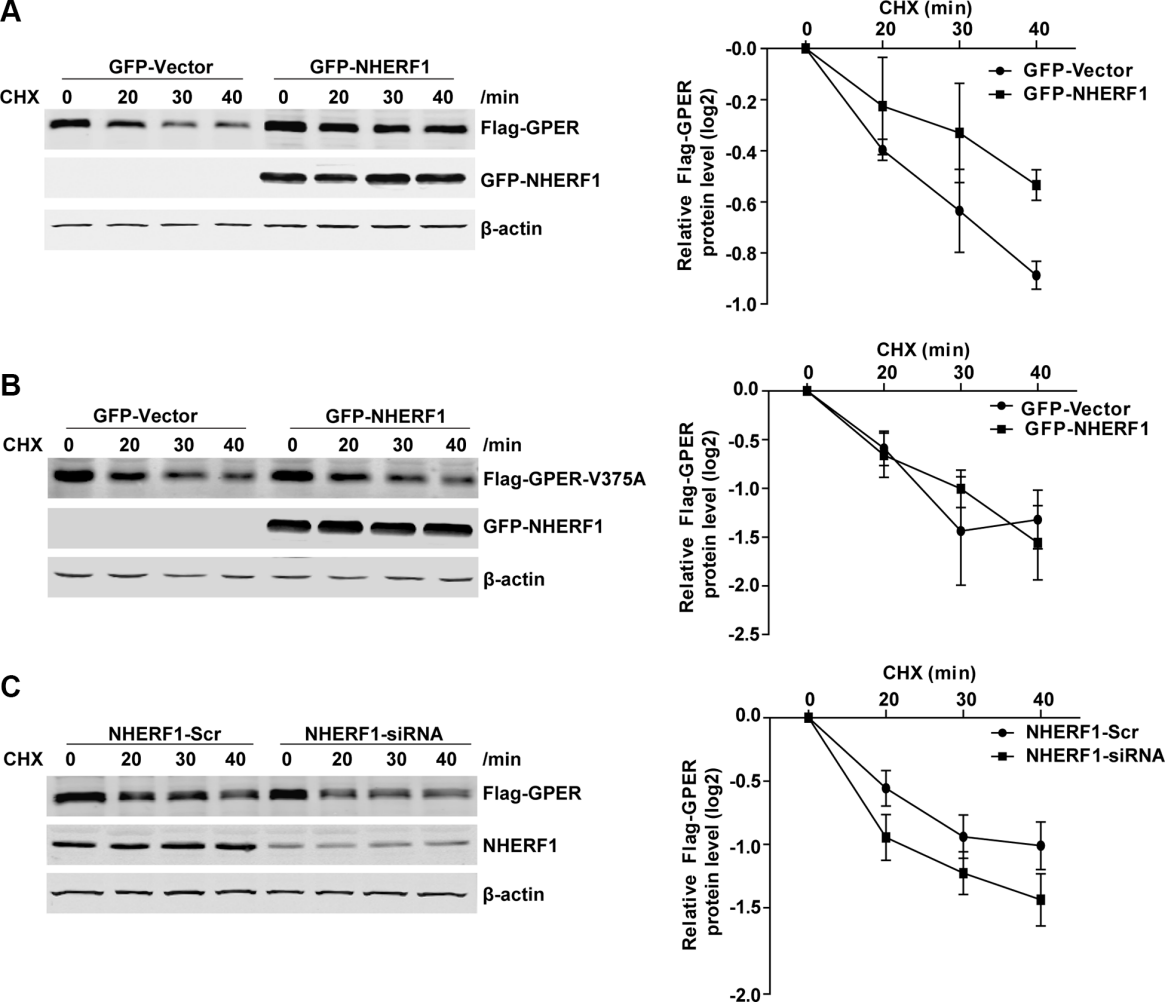
NHERF1 expression improves GPER protein stabilization (**A**) Overexpression of NHERF1 reduces the turnover rate of Flag-GPER. HEK-GPER cells were transiently transfected GFP-Vector or GFP-NHERF1, then treated with CHX (20 μg/ml) and harvested at the indicated time points. The protein levels of GPER, NHERF1, and β-actin were analyzed by Western blotting. (**B**) NHERF1 has no effect on the protein stabilization of Flag-GPER-V375A. HEK-GPER-V375A cells were transiently transfected with GFP-Vector or GFP-NHERF1, then treated with CHX (20 μg/ml) at indicated time points prior to harvest. The protein levels of GPER, NHERF1, and β-actin were analyzed by Western blotting. (**C**) Knock-down of NHERF1 expression enhances Flag-GPER degradation. HEK-GPER cells were transiently transfected with siNHERF1 or scrambled sequence (Scr). Cells were then treated with CHX (20 μg/ml) for indicated time points. Western blotting was performed to detect the levels of GPER, NHERF1, and β-actin. The experiments were repeated at least three times, with values within each experiment normalized to those of β-actin and analyzed by GraphPad Prism 5. The plot shows relative decay rates of GPER after quantified by Log_2_. Data were presented as means ± SEM.

We next explored whether NHERF1 increases GPER level by regulating GPER mRNA expression. Our results showed that although GPER protein levels varied after NHERF1 knock-down or overexpression, the GPER mRNA levels were unchanged in both cases (Figure 4D–4E). These data indicate that regulation of GPER expression by NHERF1 is not at mRNA level. NHERF1 may facilitate GPER expression at post-translational level. To test this hypothesis, we further measured the turnover rates of GPER protein after overexpression or knock-down of NHERF1. As shown in Figure 5A–5B, NHERF1 overexpression significantly reduced the turnover rate of GPER-wt, whereas had little effect on GPER-V375A. Consistently, knock-down of NHERF1 expression robustly enhanced GPER-wt degradation (Figure 5C). Taken together, the data indicate that NHERF1-mediated up-regulation of GPER protein levels is independent of *gper* transcriptional regulation and the GPER/NHERF1 interaction is required for enhanced GPER stability by NHERF1.

### NHERF1 inhibits the ubiquitin-proteasome degradation of GPER

**Figure 6:**
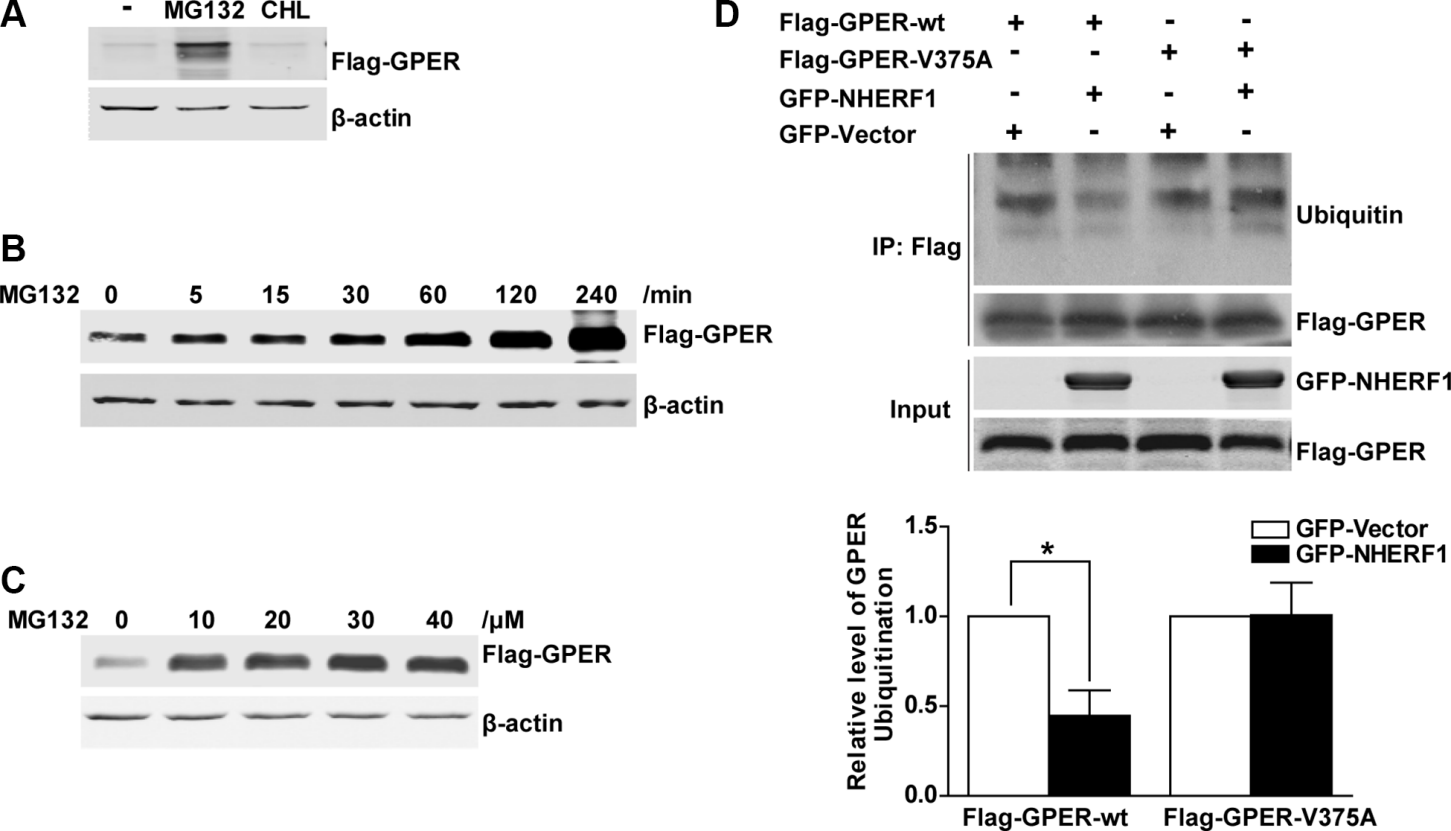
NHERF1 retards the ubiquitin–proteasome proteolysis of GPER protein (**A**) The GPER protein is degraded via the proteasome pathway. HEK-GPER cells were treated with proteasome inhibitor MG132 or lysosome inhibitor chloroquine for 4 hours. Then the GPER protein levels were detected by Western blotting. (**B**) The time course of GPER protein expression is elevated by MG132. HEK-GPER cells were treated with MG132 (20 μM) at indicated time points. The levels of Flag-GPER were examined by Western blotting. (**C**) GPER protein levels are increased by MG132 in a dose-dependent manner. HEK-GPER cells were treated with different doses of MG132 for 4 hours prior to lysis and then GPER protein levels were examined by Western blotting. (**D**) Overexpression of NHERF1 inhibits GPER ubiquitination via GPER/NHERF1 association. HEK-293 cells were co-transfected with Flag-GPER-wt or Flag-GPER-V375A in the presence or absence of GFP-NHERF1. The cells were treated with MG132 (20 μM) for 4 hours and then the lysates were immunoprecipitated using anti-Flag affinity gel. The ubiquitin levels of Flag-GPER were probed using anti-Ubiquitin antibody in the precipitate fraction. The protein levels of total Flag-GPER and GFP-NHERF1 were detected with anti-Flag and anti-GFP antibodies (lower panels).

Intracellular protein degradation occurs through the lysosomal and/or the ubiquitin-proteasome degradation pathway. To explore which pathway is involved in GPER degradation, Western blotting analysis of HEK-GPER cell lysates was performed following treatment with either the lysosome inhibitor chloroquine or the proteasome inhibitor MG132. As shown in Figure 6A, an obvious increase in GPER protein level was detected in response to the treatment of MG132, but not chloroquine, suggesting that degradation of GPER protein occurs via the proteasome pathway, which is consistent with previous study [17]. To further confirm the involvement of the proteasome degradation pathway, HEK-GPER cells were treated with MG132 at different time points and doses respectively. As shown in Figure 6B–6C, GPER protein levels significantly increased in a time and dose dependent manner when the proteasome pathway was inhibited (Figure 6B–6C).

**Figure 7:**
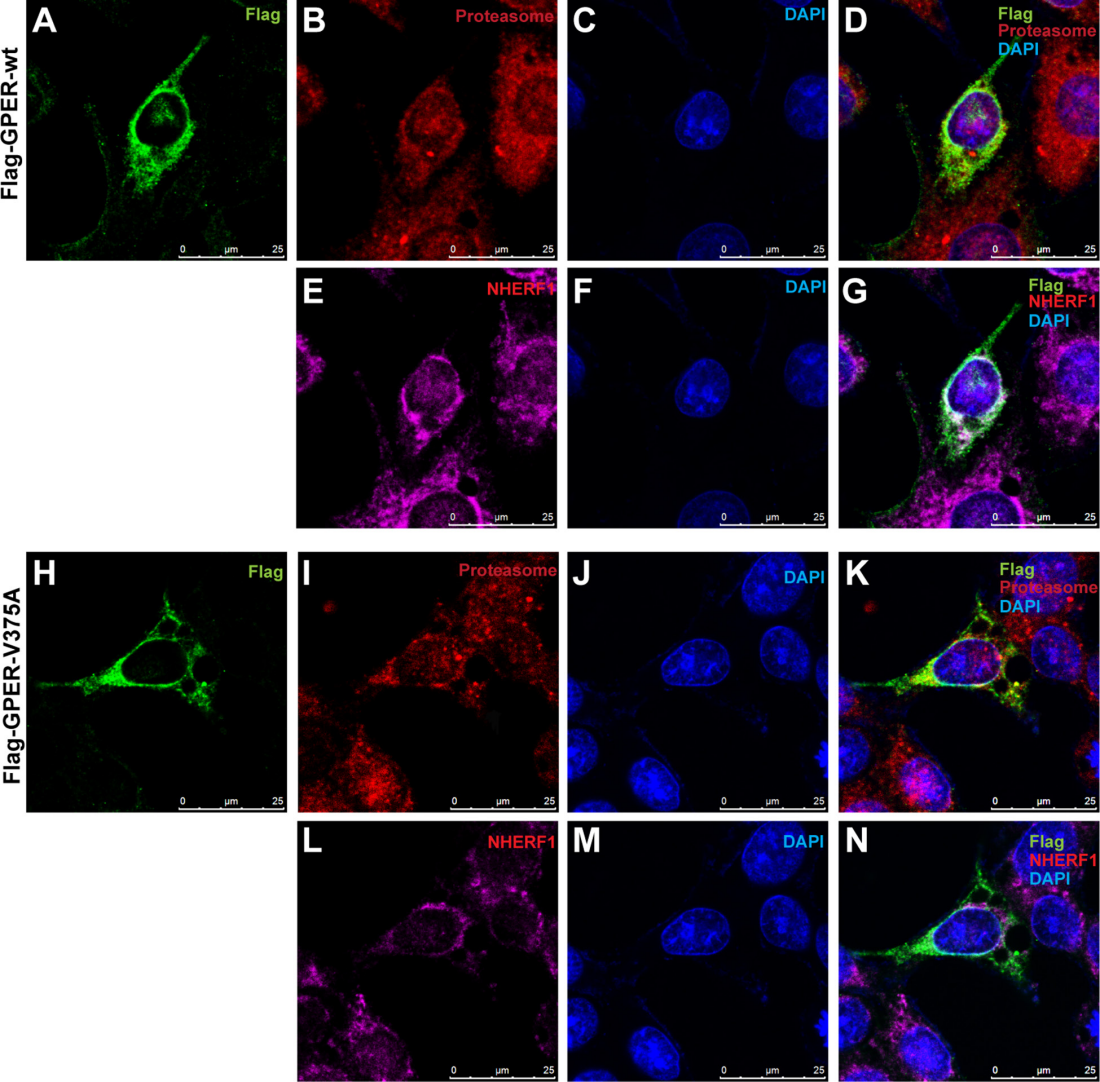
Association of NHERF1 inhibits GPER localization in the proteasome (**A**–**G**) With association of NHERF1, Flag-GPER-wt shows weak localization in proteasome. MCF-7 cells were transfected with Flag-GPER-wt. Following fixation with methanol, cells were incubated with mouse anti-Flag IgG1 (A), rabbit anti-proteasome 20 s α/β (B) and mouse anti-NHERF1 IgG2b (E) primary antibodies and then visualized using Alexa 488-conjugated anti-mouse IgG1, Alexa 594-conjugated anti-mouse IgG2b and Alexa 647-conjugated anti-rabbit IgG secondary antibodies. Nuclei were visualized with DAPI (C, F). No co-localization of proteasome and Flag-GPER-wt was detected in merged image (D). Co-localization of NHERF1 with Flag-GPER-wt was shown in panel (G). (**H**–**N**) Flag-GPER robustly co-localizes with proteasome when the interaction of GPER and NHERF1 is abolished. MCF-7 cells were transfected with Flag-GPER-V375A and then stained as described in panel (A–G). A robust co-localization of proteasome and Flag-GPER-V375A was shown in merged image (K). A little co-localization of NHERF1 with Flag-GPER-V375A was detected in panel (N).

To further verify that the regulation of NHERF1 on GPER stability is associated with the ubiquitin-proteasome pathway, we assessed the effects of NHERF1 on GPER protein ubiquitination in HEK-293 cells. As shown in Figure 6D, similar levels of Flag-GPER proteins were exhibited in all lanes detected in Western blotting, suggesting that NHERF1 has little or no effect on total GPER protein levels when ubiquitin-proteasome pathway was blocked by MG132. However, a remarkable reduction of GPER-wt ubiquitination was detected in cells co-expressed with NHERF1. In contrast, NHERF1 had little effect on the ubiquitination levels of the GPER-V375A, suggesting that GPER ubiquitination is inhibited by the interaction with NHERF1. These data were consistent with the results from our confocal microscopy experiment. When co-localized with NHERF1, GPER-wt displayed weak co-localization with proteasome 20S α/β subunits (Figure 7D, 7G). In contrast, GPER-V375A showed little co-localization with NHERF1 but marked retention in the proteasome (Figure 7K, 7N). These findings indicate that overexpression of NHERF1 increases GPER protein stability by blocking ubiquitin-proteasome degradation of GPER.

### NHERF1 positively associates with activation of GPER downstream signaling in ER-positive invasive breast cancer specimens

**Figure 8:**
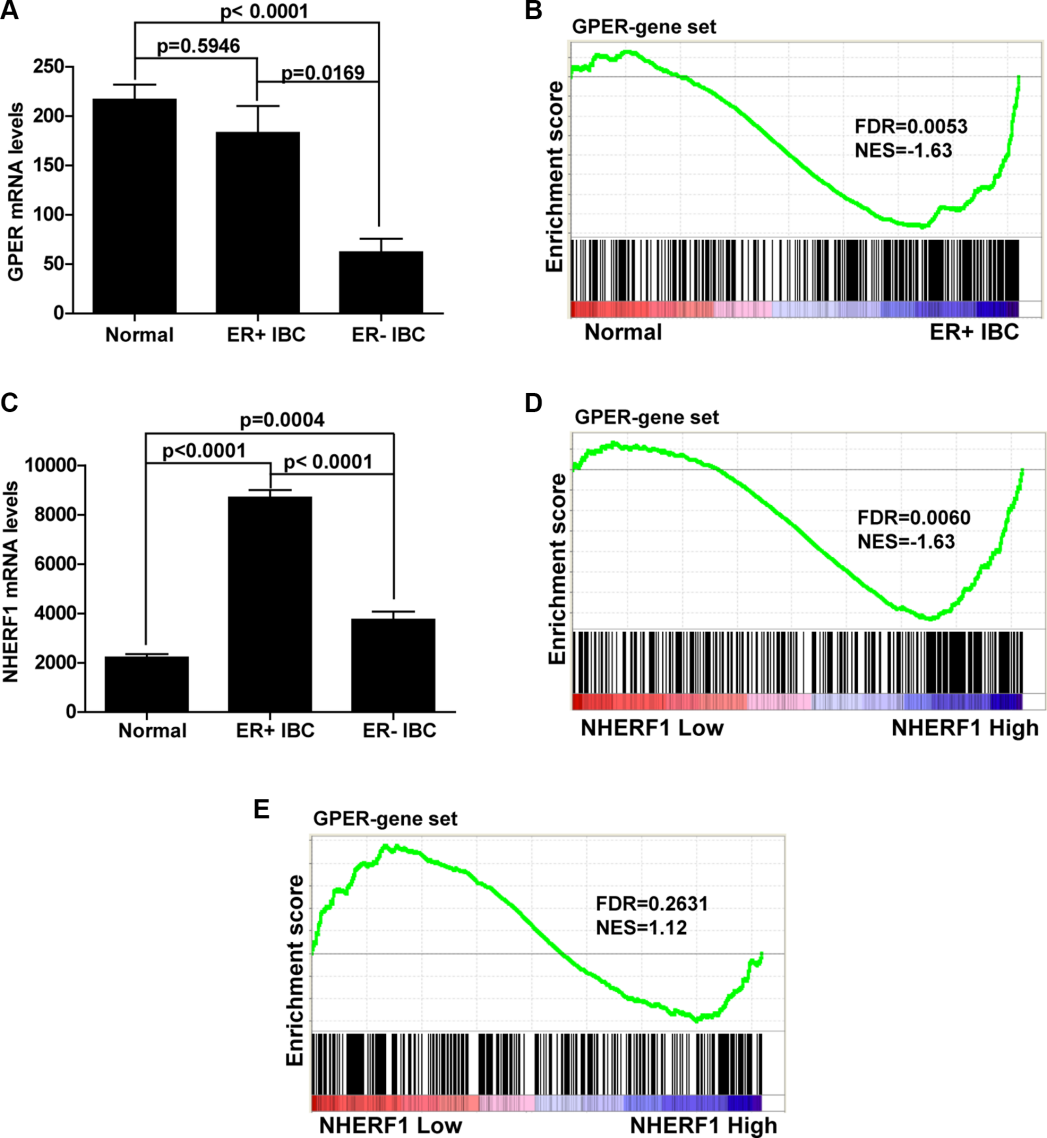
NHERF1 expression level positively associates with GPER activation in ER-positive breast cancer (**A**) The mRNA levels of GPER are similar in ER-positive invasive breast cancer (IBC) and normal samples but significantly lower in ER-negative IBC. Clinical mRNA expression data of IBC are downloaded from Sage Synapse. Significance between the 3 groups was determined with a two tailed *t*-test assuming unequal variances. (**B**) Activation of GPER downstream gene set is found in ER-positive IBC. GSEA plot for target genes of GPER (340 genes) in subgroups (Normal and ER-positive IBC) showed that genes downstream of GPER target were abundantly activated in ER-positive IBC compared with that of normal tissues. (**C**) The mRNA levels of NHERF1 are significantly up-regulated both in ER-positive and negative IBC. The mRNA levels of NHER1 were compared in the same way as GPER in panel A. (**D**) GPER downstream gene set is positively associated with NHERF1 level in ER-positive IBC. ER-positive IBC samples were divided into high and low NHERF1 expression groups. GSEA plot for target genes of GPER showed that GPER was abundantly activated in NHERF1-High subgroup compared with that of NHERF1-Low subgroup. (**E**) GPER downstream gene set is not associated with NHERF1 level in ER-negative IBC. ER-negative IBC samples were divided into high and low NHERF1 expression groups. GSEA plot for target genes of GPER showed that there was no significant enrichment of GPER gene set in neither NHERF1-High nor NHERF1-Low subgroup. False discovery rate (FDR) gives the estimated probability that a gene set with a given normalized ES (NES) represents a false-positive finding; *FDR < 0.05* is a widely accepted cutoff for the identification of biologically significant gene sets.

Based on the findings that GPER-NHERF1 interaction enhanced the stability of GPER protein, we further defined whether these findings have clinical relevance. Considering the findings by other group that GPER expression levels were different between ER-positive and ER-negative breast cancer [12], we first examined the gene expression levels of GPER in TCGA data set of invasive breast cancer (IBC) specimens. It was found that there was no significant difference in GPER mRNA levels between ER-positive IBC and normal breast tissues, whereas the GPER mRNA levels in ER-negative IBC was much lower compared with normal tissues (Figure 8A). This result indicates that regulation of GPER expression in ER-negative IBC occurred at pre-translational level. We further analyzed the activation of GPER by GSEA in normal breast tissue and ER-positive IBC specimens. As shown in Figure 8B, GPER downstream genes were mainly enriched in ER-positive IBC subgroup. This finding indicates that GPER signaling is ultra-activated in ER-positive IBC when compared with normal breast tissues, which is consistent with the high levels of GPER protein detected in clinical IBC specimens associated with poor prognosis [13, 30]. To detect whether NHERF1 was associated with this process, we next analyzed the mRNA levels of NHERF1 in normal and IBC specimens. It was found that NHERF1 mRNA levels were significantly up-regulated in both ER-positive and negative IBC, in which the levels of NHERF1 were much higher in ER-positive IBC than those in ER-negative specimens (Figures 8C, S2). To further investigate the correlation between NHERF1 expression and GPER activation in ER-positive and negative IBC, the specimens were divided into high NHERF1 and low NHERF1 groups, and further analyzed using GSEA method. As shown in Figure 8D, NHERF1 expression was found to positively correlate with the activation of GPER signaling in ER-positive IBC (Figure 8D). These data indicate that NHERF1 up-regulation in ER-positive IBC may enhance GPER stabilization and activation. In ER-negative IBC, however, there was no correlation between NHERF1 expression levels with activation of GPER signaling using the same gene set (Figure 8E).

## DISCUSSION

GPER plays an essential role in the rapid responses of estrogen and high level of GPER has been reported to associate with cancer development, especially in IBC [11]. The experiments reported here provide a novel regulatory mechanism of GPER stability by interacting with PDZ protein NHERF1, which increases GPER protein level by inhibiting GPER ubiquitin-proteasome degradation.

GPER has been reported to interact with PDZ proteins PSD95 in hippocampal dendritic spines and SAP97 in HEK-293 cells. In this study, we identified PDZ protein NHERF1 as a novel GPER-binding partner in both HEK-293 and breast cancer cells by using GST pull-down, co-IP and confocal immunofluorescent assays (Figures 1–3). Mutation of Val^375^ to Ala^375^ (V375A) in the C-terminal of GPER resulted in abolishment of this association (Figure 2C–2D, Figure 3I–3L, Figure 7N), suggesting the interaction of GPER and NHERF1 is stringent. PSD95 [31, 32] and SAP97 [33] are mainly expressed in the brain and barely expressed in breast epithelium and breast cancer cells, whereas NHERF1 is highly expressed in ER-positive breast cancer cell lines and specimens [34]. High levels of NHERF1 in breast cancer cells suggest its important roles in breast cancer. Our data identify NHERF1 as a new binding partner for GPER and provide a novel mechanism by which GPER protein level is regulated in breast cancer cells.

As an adaptor protein, NHERF1 possesses two tandem PDZ domains which can interact with different proteins. It has been reported that most GPCRs interacted with the first PDZ domain of NHERF1, such as PTHR, β2-AR, κOR and P2Y1 [35]. However, our data here show that the interaction of NHERF1 and GPER is highly dependent on the second PDZ domain of NHERF1 (Figure 2B). GPER was reported to interact with some GPCRs, such as corticotropin releasing hormone receptor-1, membrane progestin receptor-β and serotonin 5HT-1 receptor [21]. It is possible that NHERF1 may scaffold GPER with other GPCRs via its two PDZ domains and this possibility needs to be further investigated.

In the present finding, we observed that GPER protein levels were positively correlated with the levels of NHERF1 in a panel of breast cancer cells (Figure 4C). Overexpression of NHERF1 increased but knock-down of NHERF1 decreased GPER protein levels (Figure 4, Figure S1). Furthermore, we found that NHERF1 regulated GPER expression at post-translational levels by blocking GPER ubiquitin-proteasome degradation in a GPER/NHERF1 interaction dependent manner (Figure 6, Figure 7). How GPER/NHERF1 interaction regulates the ubiquitination of GPER remains elusive. Our previous studies reported that NHERF1 retarded ubiquitin-mediated proteasome degradation of PTEN by inhibiting the association between PTEN and NEDD4, an E3 ubiquitin ligase [24]. It is also possible that NHERF1 may suppress recruitment of E3 ubiquitin ligase of GPER. However, the E3 ubiquitin ligase of GPER remains unknown and this hypothesis needs to be further explored.

High expression of GPER protein was reported to correlate with poor prognosis in breast carcinoma [11]. Our result showed that the GPER signaling was robustly activated in ER-positive breast cancer specimens by GSEA from a TCGA data set (Figure 8B). These findings suggest that high levels of GPER in breast cancer may result in strong GPER activation which in turn promotes tumor progression. Meanwhile, we found no significant difference of GPER mRNA levels between ER-positive IBC samples and normal breast tissues (Figure 8A). Taken together, the above findings suggest that mechanisms contributed to GPER overexpression in ER-positive IBC may mainly occur at post-transcriptional level. In this study, we found that NHERF1 enhanced GPER protein stability at post-translational level (Figure 4). In addition, overexpression of NHERF1 was associated with aggressive clinical parameters and poor prognosis in ER-positive IBC [34, 36]. Therefore, it is likely that the elevated GPER in ER-positive breast cancer specimens might be induced by high levels of NHERF1 which further activate GPER signaling. This clinical relevance was supported by our GSEA results showing that elevated NHERF1 level was positively associated with activation of GPER signaling in ER-positive IBC (Figure 8D). Our unpublished data provide evidences that upon stimulation with GPER agonist (G-1), activation of the downstream effectors of GPER signaling such as ERK and AKT was weakened after down-regulation of NHERF in breast cancer cells. Thus, it is possible that up-regulation of NHERF1 expression may enhance the GPER stabilization and activation, which further induces ER-positive breast cancer cell proliferation and invasion. However, this hypothesis need to be further explored. It is interesting to point out that in ER-negative breast cancer, GPER protein expression was regulated not only at post-translational level by NHERF1 but also at pre-translational level. This finding indicates that mechanisms associated with the roles of NHERF1 in regulation of GPER expression may be more complicated when ER expression was absent.

As a scaffolding protein, NHERF1 is able to recruit membrane receptors/transporters and cytoplasmic signaling proteins to assemble functional complexes. However, whether NHERF1 acts as a tumor suppressor or oncoprotein in breast cancer still remains elusive. NHERF1 is reported to suppress breast cancer cell viability when it scaffolds with tumor suppressors [37], whereas it becomes an oncogenic protein when it interacts with oncoproteins [38, 39]. Information regarding how NHERF1 coordinates the formation of functional complexes to elicit its dual roles still remains limited.

In summary, our study identified a novel interaction of NHERF1 and GPER mediated by PDZ2 domain of NHERF1 with the carboxyl terminal of GPER. Association of NHERF1 improved GPER stabilization by inhibiting ubiquitin-mediated proteasome degradation of the receptor. It was further confirmed in clinical samples that NHERF1 expression level positively correlated with GPER signaling activation in ER-positive IBC. These findings provide a new insight into the regulatory mechanisms of GPER protein stability by NHERF1 in breast cancer.

## MATERIALS AND METHODS

### Cell culture and transfection

Human embryonic kidney (HEK)-293 cells and breast carcinoma MCF-7 cells were grown in Dulbecco's modified eagle media (DMEM) supplemented with 10% fetal bovine serum (FBS). Human breast cancer cell lines T47D, ZR-75-1 and SK-BR-3 were maintained in RPMI-1640 with a supplement of 10% fetal bovine serum (FBS). BT474 cells were grown in RPMI-1640 supplemented with 10% FBS and 0.01 mg/ml insulin. All cells were cultured at 37°C in a humidified incubator with 5% CO_2_. All cell culture medium and FBS were purchased from Hyclone (Logan, Utah). Lipofectamine 2000 Reagent (Invitrogen, Carlsbad, CA) was used for cell transfection. HEK-293 cells that stably express Flag-GPER or Flag-GPER-V375A were selected with the growth medium containing 1 mg/mL G418 and then maintained in growth medium containing 500 μg/mL G418 (Calbiochem, San Diego, CA).

### Antibodies

The monoclonal rabbit anti-GPER (sc-48525-R) and anti-ubiquitin (#3933S) antibodies were purchased from Santa Cruz Biotechnology (Santa Cruz, CA) and Cell Signaling Technology (Danvers, MA) respectively. The monoclonal mouse anti-NHERF1 IgG2b (MA1-19292) was purchased from Thermo Fisher (Rockford, IL), the monoclonal mouse anti-NHERF1 IgG (#611161) was purchased from BD (Franklin Lakes, NJ). The polyclonal rabbit anti-HA (#561) and anti-proteasome 20s α/β (ab22673) antibodies were purchased from MBL (Nagoya, Japan) and Abcam (Cambridge, UK) respectively. Monoclonal mouse anti-Flag (F3165) antibody was purchased from Sigma-Aldrich (St Louis, MO).

### Preparation of plasmids

The constructs encoding Flag-GPER (EX-M0792-M12) were purchased from Gene Copoeia (Guangzhou, China). The V375A mutation of Flag-GPER, the wild type HA-GPER and HA-GPER-V375A were amplified by PCR using indicated primers shown in Table 1 and then subcloned into pReceiver-M12 and pCMV-HA vectors respectively. Each plasmid was individually confirmed by DNA sequencing. pSuper.puro luciferase control (shNC) plasmid and pSuper.puro shNHERF1 (targeting the sequence CTGACGAGTTCTTCAAGAA) constructs were kind gifts from Dr. M. J. Wheelock (University of Nebraska Medical Center, Omaha, NE). pEGFP-C2 plasmids encoding full-length NHERF1 and pGEX plasmids encoding GST fusions of full-length NHERF1, NHERF1-PDZ1, NHERF1-PDZ2 and NHERF1-CT were kindly provided by Randy Hall (Emory University, Atlanta, GA).

**Table 1:** Primers used in plasmid construction

Plasmid amplified/primer	Sequence 5′- 3′
HA-GPER-wt	
Forward primer	GCGAATTCGAATGGATGTGACTTCCC
Reverse primer	TGGGTACCCTACACGGCACTGCTG
HA-GPER-V375A	
Forward primer	GCGAATTCGAATGGATGTGACTTCCC
Reverse primer	TGGGTACCCTACGCGGCACTGCTG
Flag-GPER-V375A	
Forward primer	ATCTCGAGGTCACGCTGGGCTTCATCG
Reverse primer	ATCGCCGGCGCTACGCGGCACTGCTGAAC

### Western blotting

Whole cell lysates or immunoprecipitated samples were resolved in 10% SDS-PAGE gels and transferred to PVDF membrane (Millipore, MA). After being blocked with 5% non-fat dried milk for 1 hour at room temperature, membranes were incubated in primary antibody overnight at 4°C. Horse radish peroxidase (HRP)-conjugated (ZSGB-BIO, Beijing, China) or infrared fluorescent dyes (IRDye)-conjugated (LI-COR Biosciences, Lincoln, NE) secondary antibodies were used to detect the immunoreactivity by enhanced chemiluminescence (ECL) detection reagents (Applygen Technologies, Beijing, China) and Odyssey infrared imaging system (LI-COR Biosciences, Lincoln, NE) respectively.

### GST fusion protein pull-down assay

GST pull-down assay was performed as previously described [18]. Briefly, equal amounts of GST control or GST fusion proteins beads were incubated with equal amounts of cell lysates. After incubation at 4°C for 3 hours, the samples were centrifuged to collect the beads and the beads were washed five times with ice-cold Washing Buffer (100 mM NaCl, 10 mM Hepes, pH 7.4, 5 mM EDTA, 1 mM benzamidine, 3% BSA and 0.1% Tween-20). The bound fractions were eluted from the beads using SDS-PAGE loading buffer (50 mM Tris/HCl, 100 mM DTT, 2% SDS, 0.1% Bromophenol Blue and 10% glycerol), boiled for 5 minutes, and then analyzed by Western blotting.

### Co-immunoprecipitation experiment

Co-immunoprecipitation was performed as described previously [40]. Flag-GPER and endogenous GPER were immunoprecipitated using anti-Flag affinity gel (Sigma-Aldrich) or anti-GPER antibody pre-bound to protein A/G–agarose beads (Santa Cruz Biotechnology) respectively. After the incubation at 4°C for 3 hours, agarose beads were washed with Washing Buffer for five times. Precipitated fractions were eluted using SDS-PAGE loading buffer, boiled for 5 minutes, and subjected to Western blotting analysis.

### RT-PCR

Total RNA of HEK-293 that transfected with indicated Flag-GPER (HEK-GPER) was isolated using Trizol reagent (Invitrogen, Carlsbad, CA) according to the manufacturer's instruction. The GPER and NHERF1 mRNA levels were determined using an RT-PCR kit (New England Biolabs, Beverly, MA) and primers listed in Table 2. PCR reaction mixtures were separated in 1% agarose gel and visualized under UV light. Relative GPER mRNA abundances versus β-actin mRNA were quantified.

**Table 2:** Primers used in RT-PCR

Gene primer	Sequence 5′-3′
GPER	
Forward primer	CGAGAAGATGACCATCCCCG
Reverse primer	GCTTGTCCCTGAAGGTCTCC
NHERF1	
Forward primer	AGGTCAATGGTGTCTGCA
Reverse primer	CTTTAGCCACAGCCAAGGA
β-actin	
Forward primer	GTACCACAGGCATTGTGATGGACT
Reverse primer	CTTTGATGTCACGCACGATTTCCC

### Immunofluorescence

Immunofluorescence was performed as described previously [41]. Cells on glass coverslips were rinsed with PBS for three times and then fixed with methanol for 20 minutes at −20°C. After washed for three times with PBS, primary antibodies were diluted in the blocking buffer (1% BSA in PBS) respectively and added to the coverslips for 1 hour at room temperature. After being washed for three times, coverslips were then incubated with Alexa 488/594/647-conjugated secondary antibodies (Life Technology, 1:100) for 45 minutes. After being washed for three times again, nucleuses were stained with DAPI. The coverslips were then mounted on glass slide. Immunofluorescence images were visualized with a Leica TCS SP8 confocal microscopy system (Leica Camera) with a 63X oil immersion objective.

### Cycloheximide chase assay

HEK-GPER or HEK-GPER-V375A cells were transiently transfected with GFP or GFP-NHERF1, scrambled sequence or siNHERF1 (sequences shown in Table 3), respectively. Following 24 hours of transfection, cells were treated with 20 μg/ml of cycloheximide (CHX) for 0, 20, 30, or 40 minutes respectively. The protein levels of Flag-GPER were detected using Western blotting.

**Table 3:** RNA interfere sequences used

Gene	Sequence 5′- 3′
NHERF1-siRNA	
Sense	GUCGACCACCAGCAGGCGCACGGCGUUG
Antisense	CAACGCCGUGCGCCUGCUGGUGGUCGAC
Scrambled sequence	
Sense	UCCAGACGGCGCAGUGGGCGACCGCUAC
Antisense	GUAGCGGUCGCCCACUGCGCCGUCUGGA

### Ubiquitination assay

HEK-293 cells were transfected with Flag-GPER-wt or Flag-GPER-V375A in the absence or presence of GFP-NHERF1 respectively. After 24 hours, MG132 (Sigma, 20 μM) was added and treated for 4 hours. Then cell lysates were precipitated using anti-Flag affinity gel, followed by Western blotting analysis with anti-ubiquitin antibody.

### TCGA data analysis and gene set enrichment analysis

Gene expression profile of invasive breast carcinoma (unc.edu BRCA IlluminaHiSeq RNASeqV2.geneExp) from The Cancer Genome Atlas (TCGA) database (http://cancergenome.nih.gov/) was downloaded from Sage Synapse (www.synapse.org/).

The association between gene expression and biological processes was analyzed using Gene Set Enrichment Analysis (GSEA2-2.2.1, http://www.broad.mit.edu/gsea/) [42]. GSEA calculates a pathway Enrichment Score (ES) that estimates whether genes from pre-defined gene set of GPER target genes are enriched among the highest- (or lowest-) ranked genes or distributed randomly. GPER gene set was defined as genes down-regulated with at least 10% reduction in MCF-7 cells treated with siGPER (GSE7033, dataset obtained from NIH Gene Expression Omnibus). Default settings were used and thresholds for significance were determined by permutation analysis (1000 permutations). False Discovery Rate (FDR) was calculated. A gene set is considered significantly enriched when the FDR score is *< 0.05*.

### Statistical analysis

Statistical analyses were performed using the software GraphPad Prism 5. Results are expressed as mean ± SEM. One-way analysis of variance followed by Tukey's multiple comparison test was used to determine statistical significances. Statistical significance was accepted for *p < 0.05*.

## SUPPLEMENTARY MATERIALS


