# Supplementary material for: NHERF1, a novel GPER associated protein, increases stability and activation of GPER in ER-positive breast cancer
Source: Oncotarget. 2016 Jul 19;7(34):54983–97. doi: 10.18632/oncotarget.10713 (PMC5342396; doi:10.18632/oncotarget.10713)
Supplement: Supplementary file 1 [file oncotarget-07-54983-s001.pdf]

NHERF1, a novel GPER associated protein, increases stability and activation of GPER in ER-positive breast cancer

Supplementary Materials

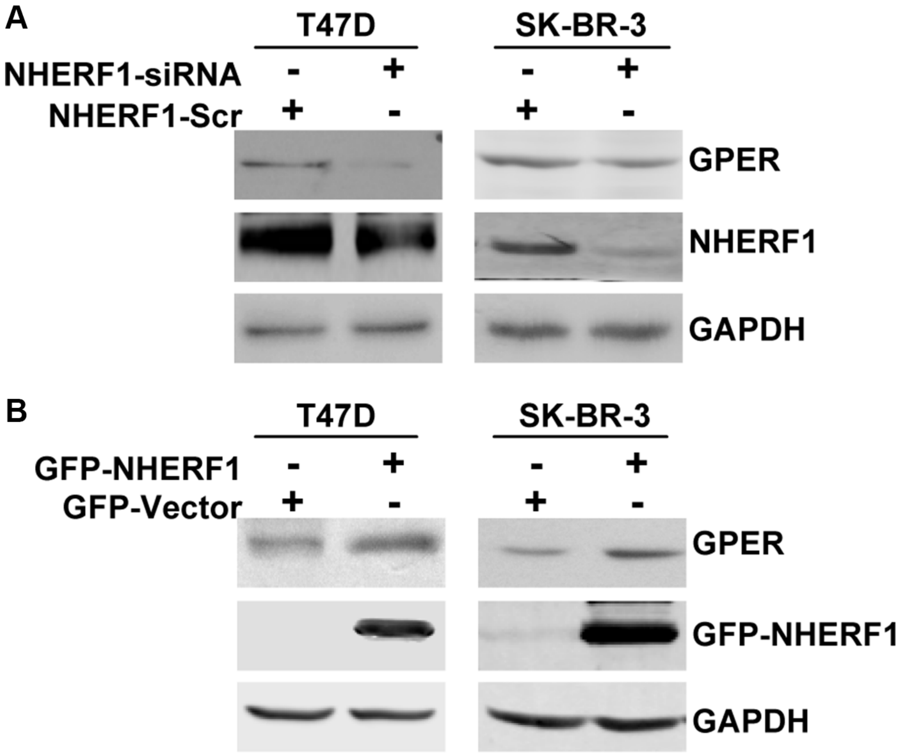

Supplementary Figure S1: NHERF1 expression enhances the protein levels of GPER in T47D and SK-BR-3 cells. (**A**) Knock down of NHERF1 expression decreases GPER protein level.T47D and SK-BR-3 breast cancer cells were transiently transfected with NHERF1-siRNA or scrambled sequence (Scr) for 24 hours. The endogenous GPER protein was then detected by Western blotting using anti-GPER antibody. (**B**) Overexpression of NHERF1 increases GPER protein level. T47D and SK-BR-3 cells were transiently transfected with GFP-Vector or GFP-NHERF1 for 24 hours. The endogenous protein of GPER was then analyzed by Western blotting using anti-GPER antibody.

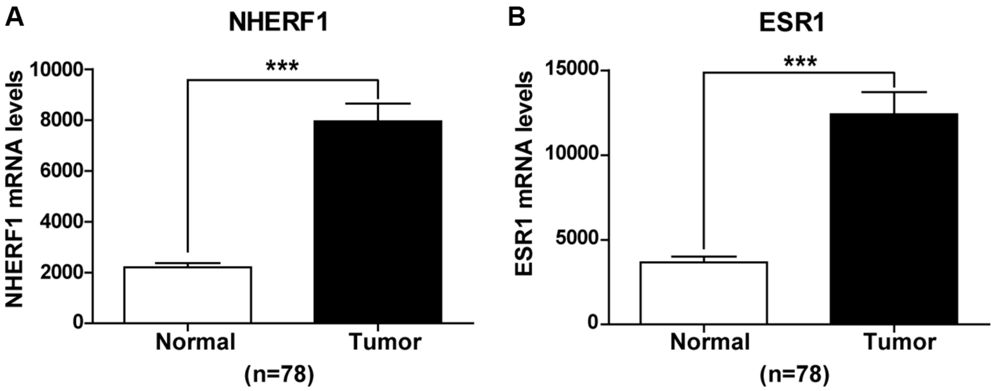

Supplementary Figure S2: NHERF1 and ESR1 mRNA levels are up-regulated in ER-positive invasive breast cancer specimens. NHERF1 (**A**) and ESR1 (**B**) mRNA levels in ER-positive breast cancer specimens and paired normal breast tissues (n = 78) were analyzed based on the data set from TCGA. The levels of NHERF1 mRNA in normal breast and breast cancer were 2205 ± 165.2 and 7944 ± 702.5, respectively. The levels of ESR1 mRNA in normal breast and breast cancer were 3670 ± 340.4 and 12400 ± 1312, respectively. The difference between the ER-positive breast cancer group and the paired non-tumor breast group was statistically significant (***$p < 0.001$).
